# Polybenzoxazole Nanofiber-Reinforced Moisture-Responsive Soft Actuators

**DOI:** 10.1038/s41598-017-00870-w

**Published:** 2017-04-10

**Authors:** Meiling Chen, Johannes Frueh, Daolin Wang, Xiankun Lin, Hui Xie, Qiang He

**Affiliations:** 1 Key Laboratory of Microsystems and Microstructures Manufacturing, Ministry of Education, Micro/Nanotechnology Research Centre, Harbin Institute of Technology, Harbin, 150080 China; 2 State Key Laboratory of Robotics and Systems, School of Mechatronics Engineering, Harbin Institute of Technology, Harbin, 150080 China

## Abstract

Hydromorphic biological systems, such as morning glory flowers, pinecones, and awns, have inspired researchers to design moisture-sensitive soft actuators capable of directly converting the change of moisture into motion or mechanical work. Here, we report a moisture-sensitive poly(*p*-phenylene benzobisoxazole) nanofiber (PBONF)-reinforced carbon nanotube/poly(vinyl alcohol) (CNT/PVA) bilayer soft actuator with fine performance on conductivity and mechanical properties. The embedded PBONFs not only assist CNTs to form a continuous, conductive film, but also enhance the mechanical performance of the actuators. The PBONF-reinforced CNT/PVA bilayer actuators can unsymmetrically adsorb and desorb water, resulting in a reversible deformation. More importantly, the actuators show a pronounced increase of conductivity due to the deformation induced by the moisture change, which allows the integration of a moisture-sensitive actuator and a humidity sensor. Upon changing the environmental humidity, the actuators can respond by the deformation for shielding and report the humidity change in a visual manner, which has been demonstrated by a tweezer and a curtain. Such nanofiber-reinforced bilayer actuators with the sensing capability should hold considerable promise for the applications such as soft robots, sensors, intelligent switches, integrated devices, and material storage.

## Introduction

Soft actuators, which can directly convert externally physical or chemical stimuli to mechanical reconfiguration and motion^1–14^, have shown great potentials in a variety of applications such as rehabilitation devices, sensors, artificial muscles, switching devices, and robotics^15–24^. Many examples in nature exhibit the humidity-sensitive behaviors that are analogous to what artificial actuators do. For instance, the petals of morning glory flowers can absorb water and expand, leading to the blooming in the morning, whereas they can fold up upon desorbing water^25^. The scales of pinecones can move to respond to the changing humidity^26^. Inspired by these humidity-sensitive natural systems, diverse carbon-based actuating materials have been employed to fabricate smart, responsive devices with different architectures such as walking robots, claws, legs, and flowers^27–29^. Carbon nanotubes (CNTs)^30, 31^ are considered to be ideal actuating materials due to their unsurpassed features such as high aspect ratios and surface area, as well as outstanding mechanical, thermal, and electrical properties. Recently, CNT-based moisture-responsive actuators, such as the graphene/CNT bilayer^32^ and the liquid crystalline hydroxypropylcellulose/CNT composite^33^, have been developed, which avoids the consumption of power and overcomes the limitation that CNT-based actuators can only be driven by the electronic stimuli^34–44^. However, the mechanical properties and the multifunctionality of moisture-responsive CNT-based actuators should be improved for the future applications.

Herein, we fabricated a moisture-responsive soft actuator with a CNT/poly(vinyl alcohol) (PVA) bilayer structure. The addition of poly(*p*-phenylene benzobisoxazole) nanofibers (PBONFs) in the CNT layers is beneficial to forming a continuous, robust film, and can effectively improve the mechanical performance of the resulting actuators. The PBO fibers, as a kind of the so-called “Super Fibers”, possess exceptionally high specific mechanical properties, thermal stability, environmental stability, and flame resistance, and have worked as important components for the reinforcement of advanced composites^45^. Due to its high flexibility and pronounced moisture-induced swelling, PVA has been employed by casting onto as-prepared PBONF-reinforced CNT layers to produce a compact double-layered structure^46^. The obtained PBONF-reinforced CNT/PVA bilayers exhibit strong mechanical properties, high flexibility, and high electric conductivity. In consequence of the unsymmetrical absorption of water in the CNT/PVA double-layered structure, the actuators exhibit the actuation behavior with large deformation, precisely controlled direction, and good durability. More importantly, the electric conductivity of the actuators is highly sensitive to the variation of relative humidity (RH), and increases along with the augment of RH, which is quite different from that of the CNT/PVA monolayer^47, 48^. The PBONF-reinforced CNT/PVA actuators can be used to construct smart, multifunctional devices demonstrated by an electronic tweezer and a curtain, indicating the potential applications of the soft actuators in various fields such as humidity sensors, artificial muscles, and moisture-proof equipment.

## Results and Discussion

**Figure 1:**
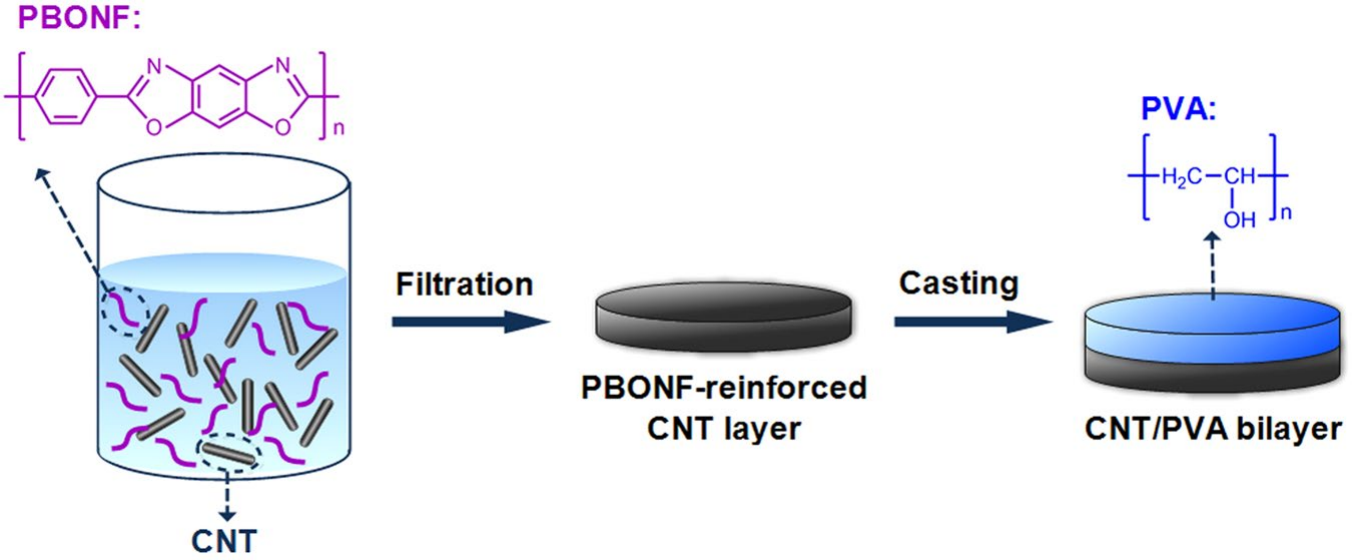
Schematic illustration of the fabrication procedure for the PBONF-reinforced CNT/PVA bilayer actuators.

**Figure 2:**
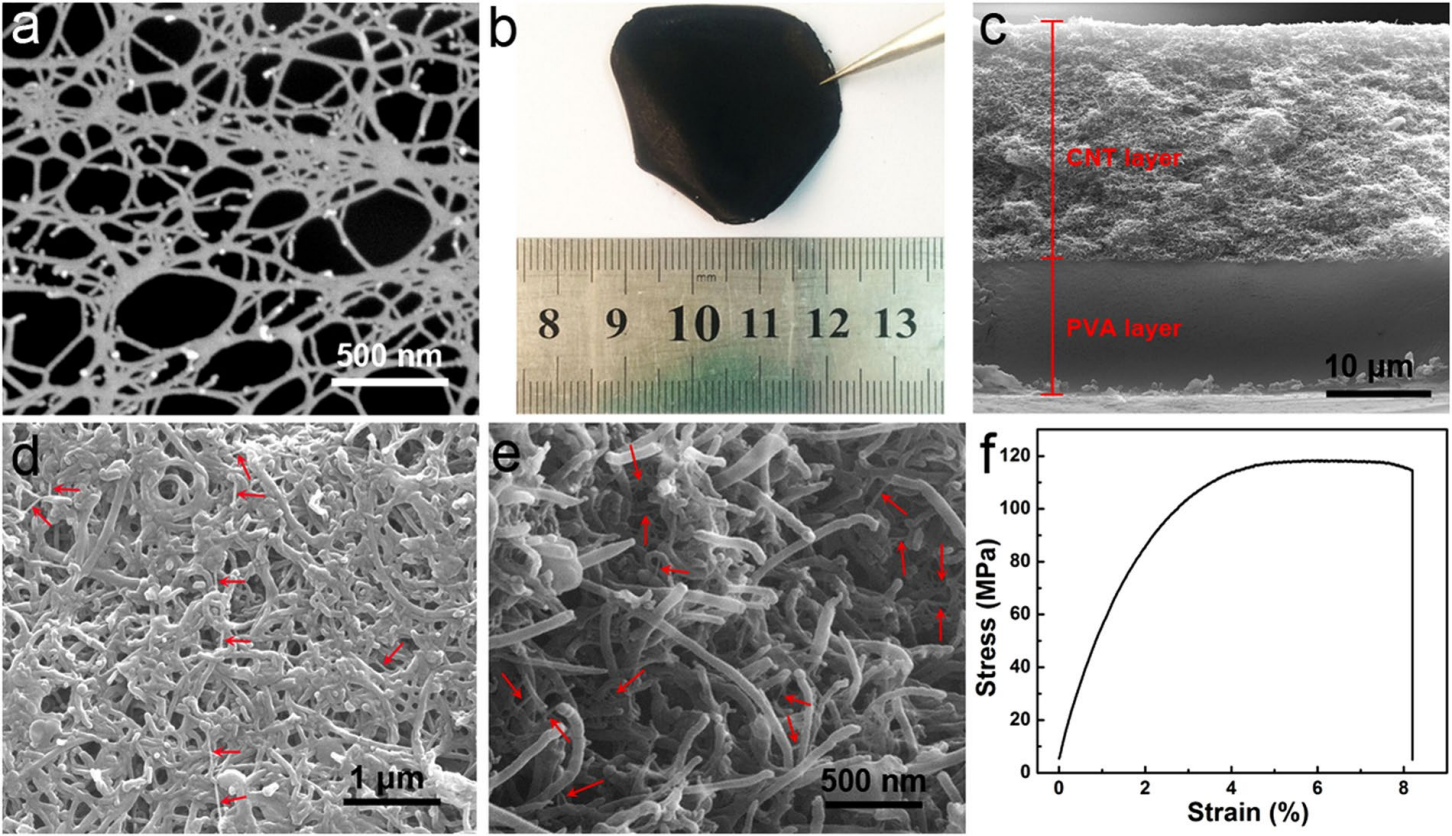
Structure characterization of the PBONF-reinforced CNT/PVA bilayer actuators. (**a**) SEM image of PBONFs. (**b**) Photograph of a PBONF-reinforced CNT/PVA bilayer film with the diameter of ca. 4 cm. (**c**) Cross-sectional SEM image of the bilayer actuator. (**d**) Top-view and (**e**) cross-sectional SEM images of the PBONF-reinforced CNT layer. Red arrows mark PBONFs distributed in the CNT network. (**f**) Tensile stress-strain curve of the PBONF-reinforced CNT/PVA bilayer actuator at the RH of 32%.

As shown in the schematic illustration in Fig. 1, the PBONF-reinforced CNT/PVA bilayer actuators can be conveniently fabricated by a vacuum-assisted method. PBONFs were firstly prepared by treating PBO fibers with the mixed acids according to the previous method^49^. Both scanning electron microscopic (SEM) image in Fig. 2a and transmission electron microscopic results (Fig. S1) confirm that PBONFs with the diameter of ca. 20 nm have been successfully synthesized. The PBONF-reinforced CNT layer was produced by vacuum filtering of the CNT and PBONF suspension (PBONF:CNT = 1:5 by weight) through a 0.45 μm polytetrafluoroethylene filter membrane. After that, the PVA solution (3 wt%) was deposited onto the PBONF-reinforced CNT layer through casting. The PBONF-reinforced CNT/PVA bilayer films were obtained after rinsing with water and peeling from the filter membrane. The photograph in Fig. 2b shows a freestanding PBONF-reinforced CNT/PVA bilayer film with a diameter of ca. 4 cm. It is worth to note that it is very difficult to form a continuous and freestanding film by using the CNT dispersion without PBONFs. The cross-sectional SEM image in Fig. 2c reveals that the thickness of the PBONF-reinforced CNT layer and the PVA layer is 26.5 and 15 μm, respectively. The top-view and cross-sectional SEM images (Fig. 2d,e) show that the PBONF-reinforced CNT layer forms a continuous, uniform film. The employed CNTs have a diameter of 30–50 nm. Thus, the PBONFs can be distinguished from the CNTs in the SEM image by their sizes. The embedded PBONFs have been marked by red arrows in the images. The tensile stress-strain curve in Fig. 2f reveals the strong mechanical properties of the PBONF-reinforced CNT/PVA bilayer film at the RH of 32%. The tensile strength and the strain of the bilayer film are 118.46 MPa and 8.20%, respectively, which exhibits the appropriate flexibility and the excellent strength for high-performance actuators. To our best knowledge, the tensile strength of PBONF-reinforced CNT/PVA bilayer has outclassed the maximum stress of polymer artificial muscles (ca. 35 MPa)^50^ that are triggered by electrochemical or thermal stimuli, and even exceeded the strength of a mechanically robust actuator that is driven by both moisture and light (ca. 100 MPa)^51^. Moreover, the tensile strength is 338 times higher than that of mammalian skeletal muscles (0.35 MPa)^3^. The tensile modulus of as-prepared bilayer actuators is calculated to be 5.67 GPa, which is ca. 38 times higher than that of the pristine PVA film (ca. 0.15 GPa)^49, 52^. The reinforcement should be ascribed to the fact that the polyaromatic structure of PBONFs could favor the binding of the nanofibers with CNTs through π-π interactions^53^. Hence, the PBONF-reinforced CNT/PVA bilayer actuators possess outstanding mechanical performance.

**Figure 3:**
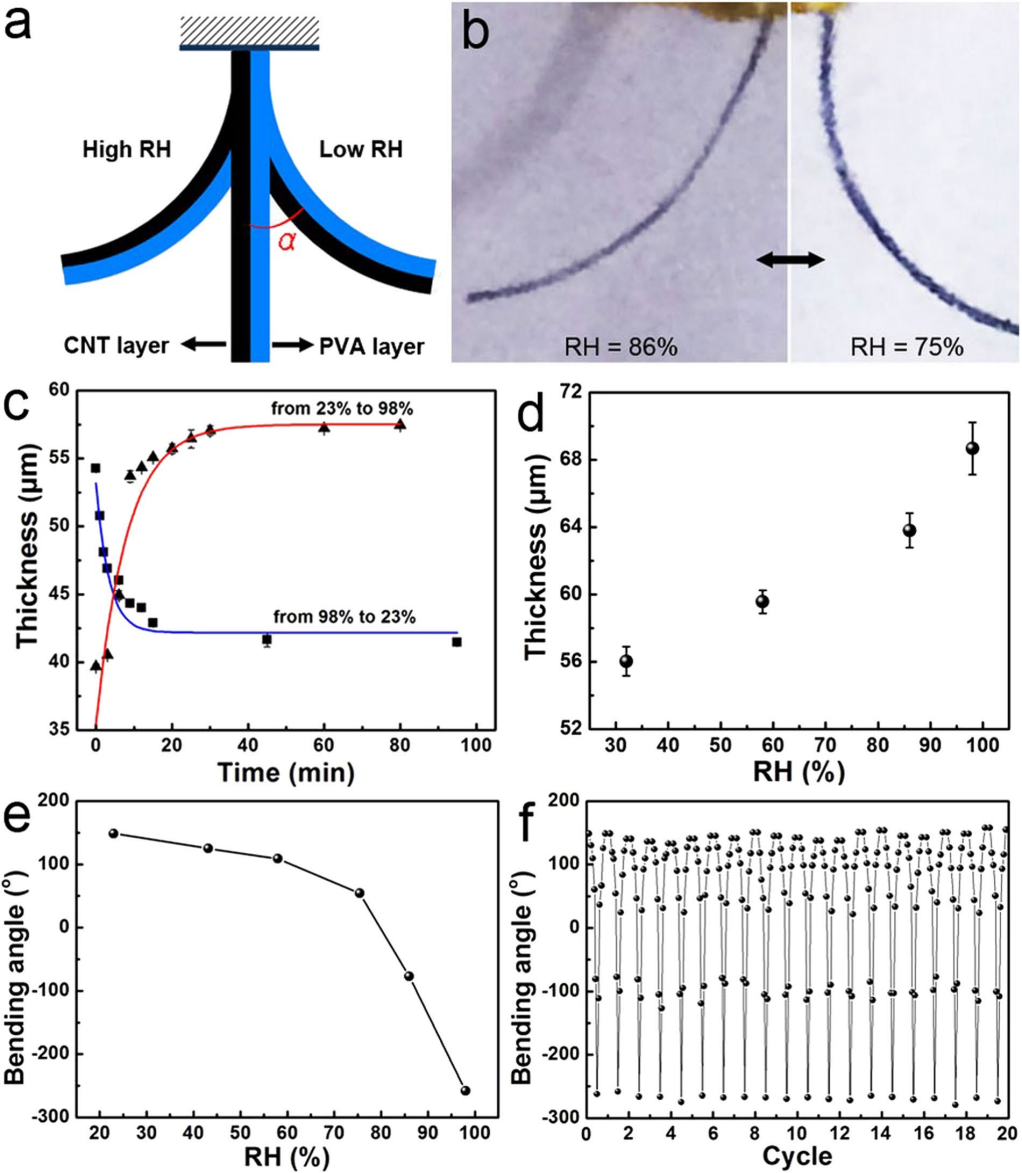
Moisture-responsive properties of the PBONF-reinforced CNT/PVA bilayer actuators. (**a**) Scheme indicating the definition of the bending angle *α*. (**b**) Photographs of the PBONF-reinforced CNT/PVA bilayer actuator at RH = 75% and RH = 86%. (**c**) Dependence of the thickness of the PVA layer on time when RH switches from 23% to 98% (red curve), and from 98% to 23% (blue curve). Two curves are added for guiding the eyes. (**c**) Thicknesses of the PVA layer at different RHs. (**e**) Bending angle of the actuator as a function of RH. (**f**) Reversible deformation of the actuator when the RH is repeatedly changed for 20 cycles.

The bending angle of the bended PBONF-reinforced CNT/PVA bilayer actuators at the different RHs could be represented by the bending angle (*α*) as shown in Fig. 3a. In this study, a strip of freestanding bilayer actuator was fixed at one end. The strip keeps a linear shape at the RH of 80%, and we defined that the angle *α* is equal to zero at this state. Moreover, the angles are negative at the situations with RH higher than 80% and positive when RH is below 80%. Photographs in Fig. 3b show that the bilayer actuator bends to the direction of the PBONF-reinforced CNT layer due to the swelling of the PVA layer at the RH of 86%, and the opposite scenario is observed at the RH of 75%. To understand the moisture-responsive mechanism of the bilayer actuators, it is necessary to measure the thickness change of the PVA layer along with the variation of RH. The swelling/shrinkage of the PVA layer should be crucial to the moisture-responsive bending of the PBONF-reinforced CNT/PVA bilayer actuators. An Olympus fluorescence microscope was used to characterize the expansion and contraction of the PVA layer. Figure 3c presents the thickness changes of the PVA layer in an actuator when the environmental RH is varied. Two curved lines are added for guiding the eyes. When RH was switched from 23% to 98%, the thickness gradually increased from 40 to 57 μm, and became almost constant after 30 min. Upon switching RH from 98% to 23%, the thickness decreased from 54 to 41 μm in 20 min. Figure 3d shows the thickness of the PVA layer at different RHs. The thickness goes up gradually from 56 to 69 μm along with the increase of RH from 32% to 98%. To study the relationship between the actuator bending angle and RH, the tested actuators were kept in the environment with the specific RH for at least 30 min before the measurement of the bending angle. Water has a much stronger affinity with the PVA layer than the PBONF-reinforced CNT layer. Therefore, upon changing RH, the PVA layer swells/shrinks markedly, whereas the thickness of the CNT layer remains almost unchanged, which results in the moisture-responsive bending of the bilayer actuators.

As shown in Fig. 3e, the PBONF-reinforced CNT/PVA bilayer actuator bends from *α* = 149° to *α* = −258° when the RH increases from 23% to 98%. The durability of the bilayer actuators is tested by changing RH repeatedly for 20 cycles. In every cycle, RH increases from 23% to 98%, and then changes back from 98% to 23%. Figure 3f shows that the moisture-responsive performance of the actuators remains reversible and stable for 20 cycles, suggesting the good durability of the actuators for long-term operation. The large range of the bending degree over 407° and the good durability for the cyclic actuation indicate that the PBONF-reinforced CNT/PVA bilayer actuators possess a highly reliable actuation performance. As shown in Fig. S2, the bilayer actuators are also sensitive to temperature. Upon elevating temperature, the PVA layer loses water, and the actuator shrinks towards the side of the PVA layer. At the RH of 30%, the bending angle *α* of the actuator increases from 141° to 168° when the environmental temperature increases from 20 °C to 50 °C, and decreases back to 155° after cooling from 50 °C to 20 °C.

**Figure 4:**
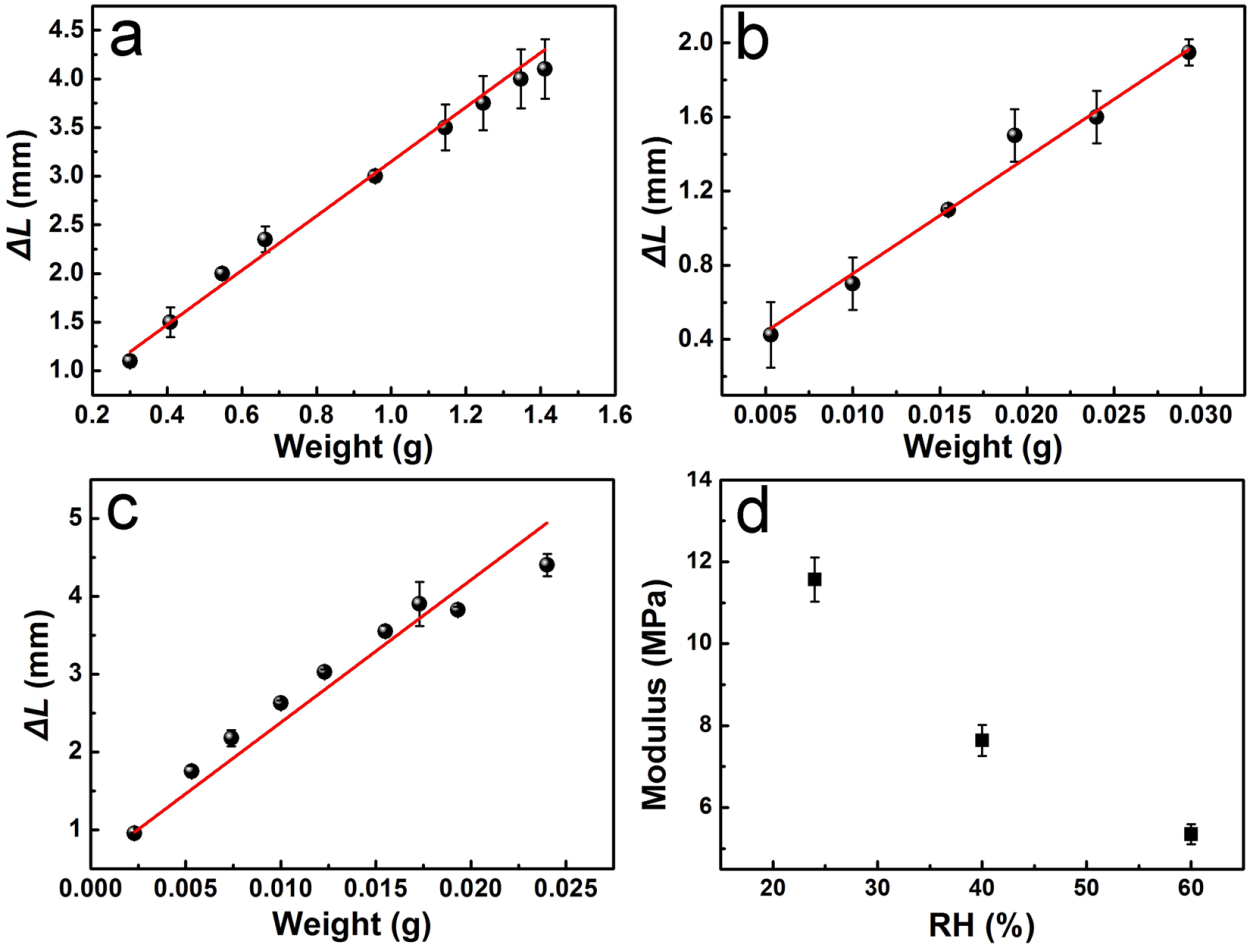
(**a**–**c**) Dependence of the moving distance of actuators (*ΔL*) on the mass of the weight that hangs on to the actuator when the RH is (**a**) 36%, (**b**) 75%, and (**c**) 90%. (**d**) Compressive modulus of the PVA layer at different RHs measured by AFM.

Due to the moisture-responsive bending, the PBONF-reinforced CNT/PVA actuators can sustain certain weights at different RHs. Aluminum foils with various mass were used as weights, and hanged on to the curled CNT/PVA actuators in the environment with a specific humidity. The moving distance of the actuator (*ΔL*) caused by the weight was measured (Fig. S3). As shown in Fig. 4a–c, when *ΔL* is 2 mm, the loaded mass is 0.58, 0.029, and 0.008 g at the RH of 36%, 75%, and 90%, respectively. This result indicates that the actuators can hold much bigger mass under the dried conditions. At the RH of 36%, the mass of the sustained weight (1.41 g) is 1281 times higher than that of the actuator itself (1.10 mg). The change of *ΔL* with the mass of the weight and the RH provides the necessary information about the elongation degree of the actuators at different RHs for the following simulation study. The influence of RH on the compressive modulus of the PVA layers in the bilayer actuators has been investigated by using a home-built atomic force microscopy (AFM). As shown in the Fig. 4d, the compressive modulus of the PVA layers decreases along with the increasing of the RH, which should be attributed to the plasticizing and hydration effect of water molecules on the hydrophilic PVA matrix^54, 55^.

**Figure 5:**
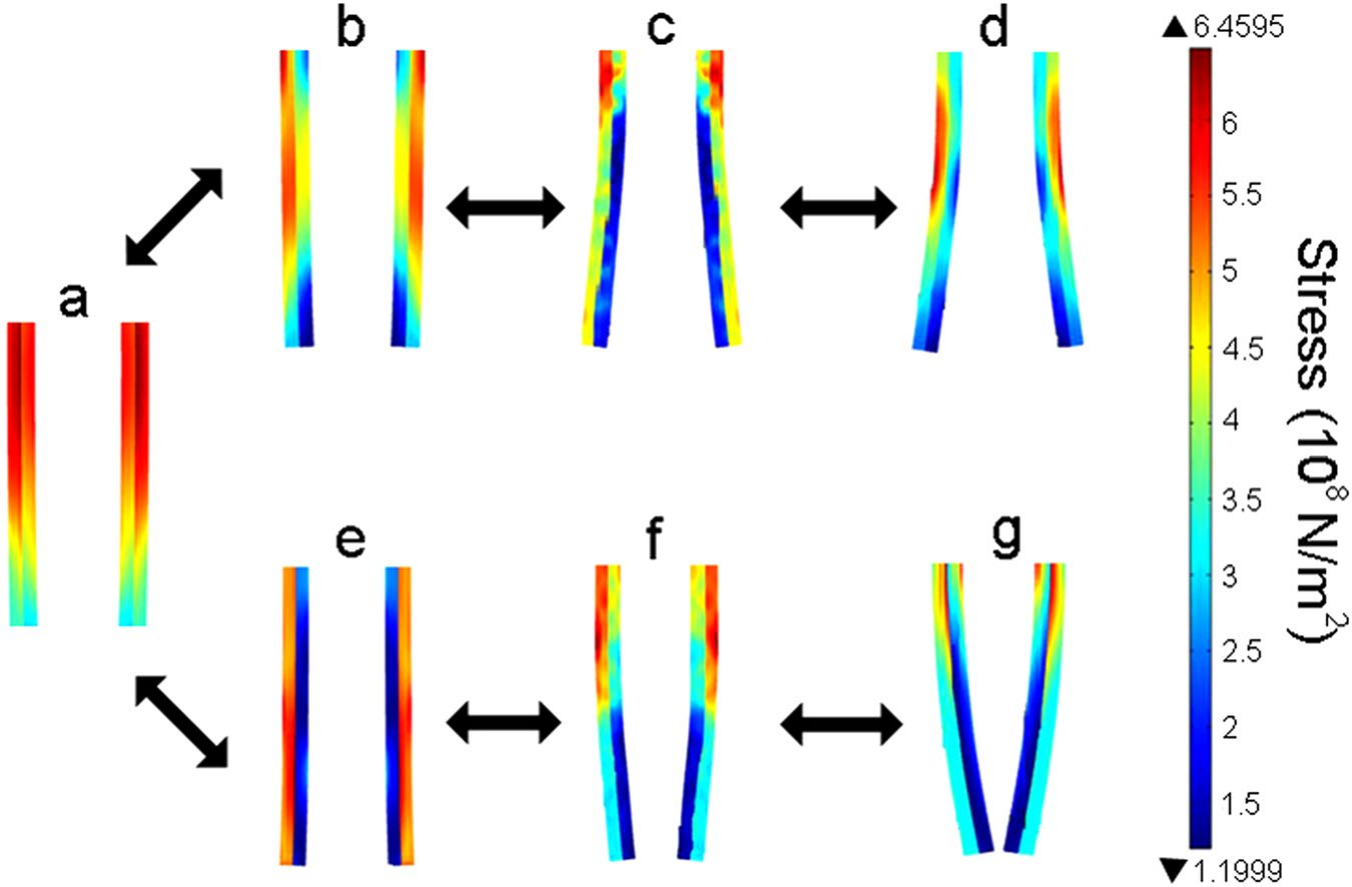
Snapshots of the finite element simulation of the bending process and the stress distribution of PBONF-reinforced CNT/PVA bilayer actuators when switching RH from 80% (**a**) to 20% (**b**–**d**) or from 80% (**a**) to 98% (**e**–**g**).

To understand the deformation mechanism of moisture-responsive bilayer actuators, finite element simulation was performed by using COMSOL Multiphysics software. To simplify the simulation of mechanical and swelling properties of the PBONF-reinforced CNT/PVA actuators, a bilayer model was used. In the simulation, the bilayer actuators were set with the upper end fixed at a constant distance and the distal end capable of friction-free sliding. The outer and inner layers were PVA layers and PBONF-reinforced CNT layers, respectively. Different colors indicate the magnitude of stress on the bilayer actuators. The simulation results in Fig. 5 verify that the gradient of the stress distributed on the bilayer structures leads to the bending of the actuators. At the original state (Fig. 5a, RH = 80%), the most stable configuration of the two actuators is the straight bilayers standing in parallel due to the homogeneous distribution of stress. Stress mechanical theory suggests the high stress zone tends to deform in order to reduce its internal stress until it reaches a balance with the low stress zone. For RH = 20%, the two free ends of the two bilayer actuators separate wider, both the internal and outer layers of soft actuators reach a minimal stress state (Fig. 5b–d). While for RH = 98%, the minimal stress state only appears when the two free ends of the two bilayer actuators get closer (Fig. 5e–g). The simulation results are consistent with the experimental data where the bilayer actuators bend towards the PVA layer at the RHs below 80%, and curl towards the CNT layer at higher RHs (>80%).

**Figure 6:**
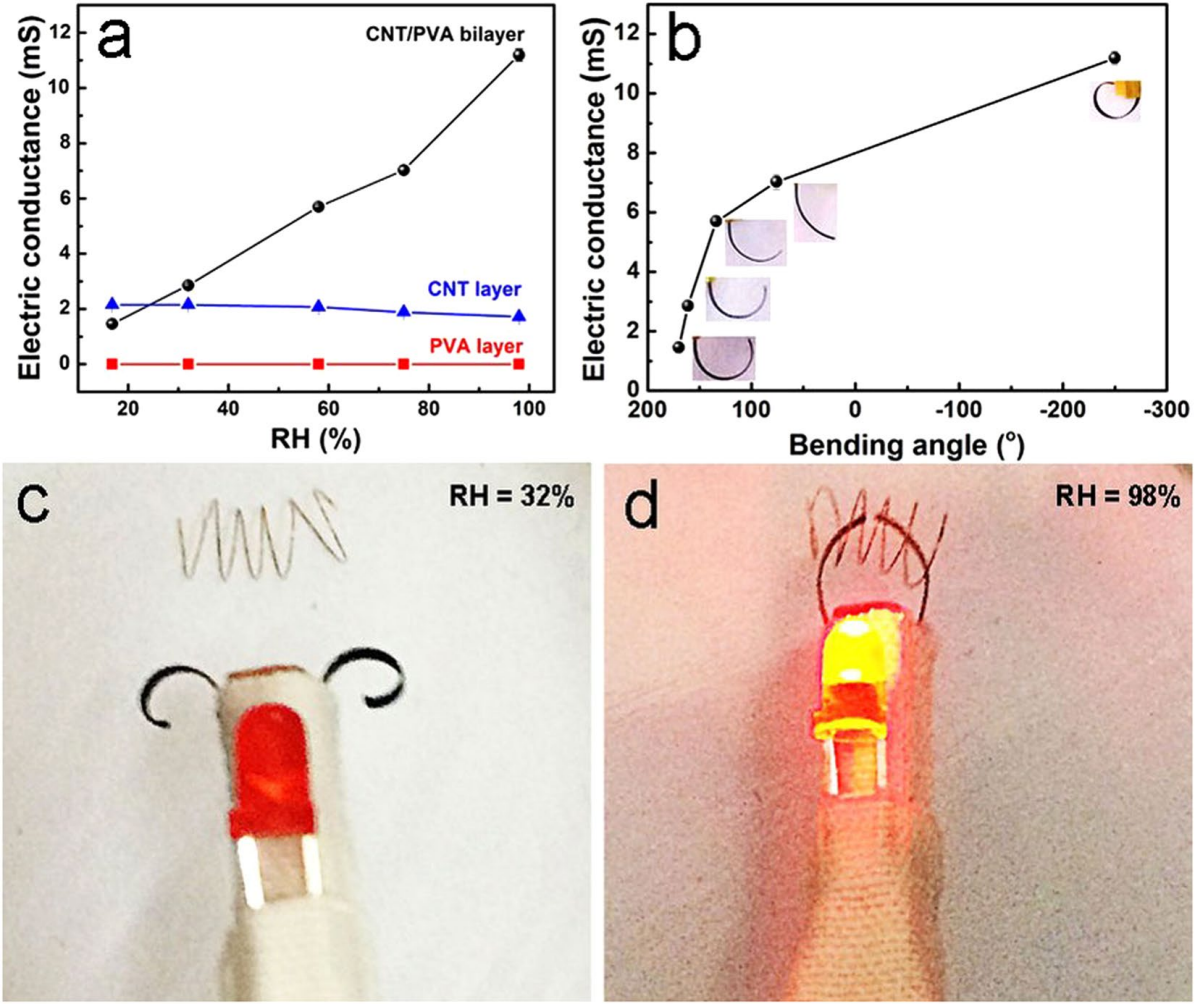
Moisture-sensitive conductivity of the PBONF-reinforced CNT/PVA bilayer actuators and its application as an electrical tweezer. (**a**) Electric conductance of the PVA layer, the PBONF-reinforced CNT layer, and the PBONF-reinforced CNT/PVA bilayers at different RHs. (**b**) Relationship between the conductance and the bending angle of bilayer actuators. Insets are photographs showing the bending degree of actuators. (**c**,**d**) Photographs illustrating that an electrical tweezer grips a copper wire, closes the circuit, and lightens up the LED bulb at the same time, when the RH is switched from (**c**) 32% to (**d**) 98%. The tweezer consists of two PBONF-reinforced CNT/PVA strips.

We studied the electric properties of the actuators. Comparing with the pristine CNT layers, the addition of PBONFs in the PBONF-reinforced CNT layers does not decrease the electric conductivity dramatically due to the low content of PBONFs (Fig. S6). As seen in Fig. 6a, the pristine PVA film with the dimension of 11.28 mm × 3.95 mm (length × width) was electrically insulated with the conductance of 9.9 × 10^−8^ mS. The conductance of the PBONF-reinforced CNT layer (7.42 mm × 3.60 mm) decreases from 2.16 to 1.72 mS when RH changes from 16.8% to 98%. Water molecules seem to play a current blocking role in the CNT layers. However, undergoing the same humidity variation, the conductance of the PBONF-reinforced CNT/PVA bilayer with the dimension of 9.28 mm × 4.39 mm and the thickness of 41.5 μm vastly increases from 1.46 to 11.2 mS, which means that the electric conductivity of the bilayer grows from 0.74 to 5.70 S/cm. To find out the reasons for this phenomenon, the control experiments have been done. First, the ionization of PVA in water was excluded, since the conductance of PVA solutions keeps constant along with the change of the voltage from 2.5 to 4 V (Fig. S4). Moreover, the conductance of the PBONF-reinforced CNT/PVA bilayer (10.00 mm × 6.00 mm) that was forced to be unbending by fixing the ends has been detected at varied RH. The conductance remains almost unchanged at three different RHs (16.8%, 58%, and 98%) (Fig. S5). Thus, the deformation of the bilayer structures along with the increased humidity should be the primary reason that leads to the increase of the conductance. The swelling of the PVA layer along with the increasing RH makes the bilayer bend to the PBONF-reinforced CNT layer, which leads to the compression of the CNT layer. This compression makes the network of CNTs more compact, which would increase the conductivity of the bilayer actuators^47, 48^. The relationship between the deformation and the conductance of the actuators has been shown in Fig. 6b. Due to their capability for converting the variation of environmental RH to the change of the electric conductance, the PBONF-reinforced CNT/PVA bilayers have great potential to act as a humidity sensor. Combining the conductivity with the moisture-responsive actuation, the PBONF-reinforced CNT/PVA bilayers can be employed to construct multifunctional devices, as demonstrated by a moisture-responsive “electrical tweezer”. A battery-powered circuit was installed, with the electrical tweezer in series with a light emitting diode (LED) bulb. The electrical tweezer was consisted of two strips of the bilayer actuators with the PVA layers as the outer layers. Figure 6c,d shows that the tweezer is able to grip a short copper wire and switch the circuit on, induced by changing the environmental humidity. When the RH was 32%, the electrical tweezer was at the open state and the circuit was also open (Fig. 6c). However, when the RH increased to 98%, the two strips of the tweezer bended in the opposite direction and gripped the copper wire, making the circuit closed and the LED bulb lighten up (Fig. 6d).

**Figure 7:**
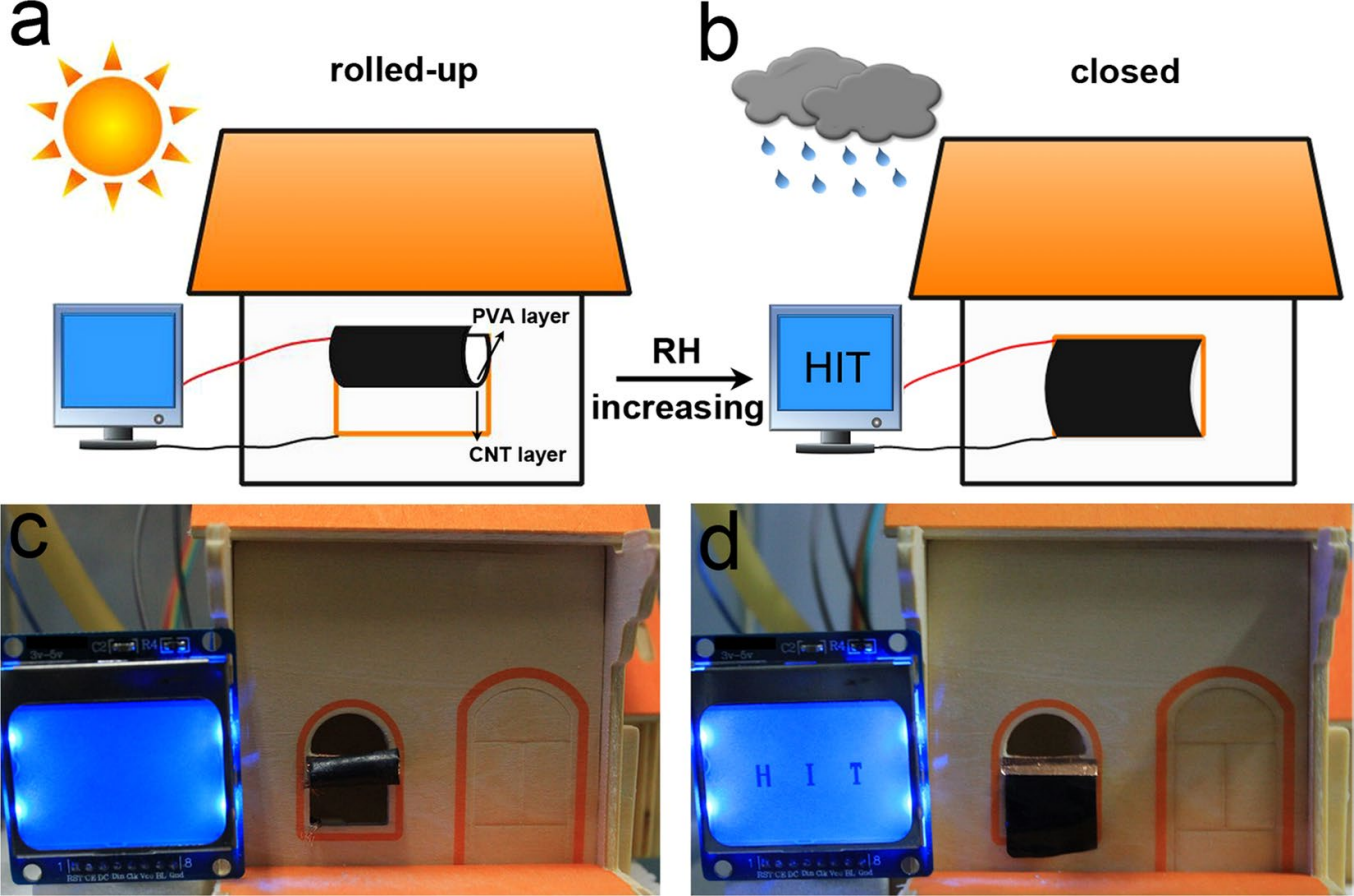
Moisture-responsive performance of a smart curtain based on the PBONF-reinforced CNT/PVA bilayer actuators. (**a**,**b**) Schematic of the setup of the smart curtain and its humidity-responsive behaviors. (**c**) The smart curtain rolls up at the RH of 32%. (**d**) When the RH is turned to be 98%, the curtain closes automatically, and the LCD displayer shows a signal of “HIT” at the same time.

Based on the PBONF-reinforced CNT/PVA bilayer actuators, a smart curtain has been designed as a demonstration. The curtain can respond to the moisture change by the deformation for shielding, and report the change in a visual manner at the same time. As seen in the schematic view in Fig. 7a,b, the demonstration system includes a bilayer actuator with the dimension of 15 mm × 16 mm (length × width), a model house with a window, and a liquid-crystal displayer (LCD) with the accessorial circuit. The curly actuator-based curtain was fixed onto the upper edge of the window frame of the model house, with the PVA layer upward and the PBONF-reinforced CNT layer downward. The LCD was connected to the upper left corner of the CNT layers of the curtain by a cable. When the fine weather was mimed and the RH around the house was 32%, the smart curtain kept a rolled-up state (Fig. 7c). However, when the weather changed to be moist and the RH became to be 98%, the PVA layer adsorbed water and swelled, but the thickness of CNT layer kept constant. As a result, the bilayer actuator gradually bended to the CNT layer, and the curtain displayed a closed state and shielded the window. At the same time, the bottom edge of the curtain touched another cable that also connected to the LCD. Owing to the high conductivity of the CNT layer in the curtain, the circuit of the LCD system was closed, and displayed a signal of “HIT” to warn of the moist environment (Fig. 7d). As shown by this example, the actuator-based curtains can be potentially applied as smart moisture-proof devices for the storage of the materials and devices that are susceptible to moisture in both warehouse and outdoor. The stored objects may include medicament, gunpowder, military equipment, electronic equipment, and so forth^56, 57^. Upon the environmental humidity increasing, the smart curtains can not only shut down and shield the goods and materials, but also supply a signal to conservators for taking measures in time.

In conclusion, we have developed a robust, smart soft actuator based on the PBONF-reinforced CNT/PVA double-layered structures through taking advantage of hygroscopic capability of PVA, mechanical performance of PBONFs, and electrical properties of CNTs. Due to the introduction of PBONFs, the actuators exhibit mechanically robust. The actuators are highly sensitive to moisture, as shown by the reversible, durable, and robust humidity-driven actuation with large deformation. Moreover, the CNT/PVA bilayer actuators are able to sense the humidity by employing the deformation-induced change of conductivity. Through integrating the inherent electric conductivity with the hygromorphic properties, the bilayer actuators can be assembled into smart devices as demonstrated by an electrical tweezer and a curtain. Note that the high performance of PBONFs such as thermo-oxidative resistance, superior mechanical tenacity, and even optoelectronic properties^45, 58, 59^, robust and multifunctional actuators are expected in the further device optimization. This study paves a way to design and construct smart moisture-responsive actuators with potential applications in diverse fields such as sensors, artificial muscles, switches, and tissue engineering.

## Methods

### Materials

The multi-walled carbon nanotubes (diameter = 30–50 nm, length = 0.5–2 μm, electrical conductivity: >100 S/cm) were received from Beijing Dk Nano technology Co., Ltd.. Trifluoroacetic acid (TFA) and methanesulfonic acid (MSA) were purchased from Aladdin reagent company. The PBO fibers (type: AS) were obtained from Toyobo Co. Ltd., Japan. A Purelab ultrapure water system (ELGA Purelab, UK) was used to produce ultrapure water (18 MΩ cm). Glass sand core filter was purchased from Beijing InnoChem Science & Technology Co., Ltd. Poly(vinyl alcohol) (molecular weight = 77000 ± 2200, purity = 97%) was obtained from Tianjin BASF Chemical Co., Ltd.

### Preparation of PBONF-reinforced CNT/PVA bilayer actuators

As reported previously, PBONFs can be obtained by treating the PBO fibers with the mixed acid of MSA and TFA^49, 60^. The commercial PBO fibers (0.02 wt%) were add into a 9.3:0.7 (v/v) mixture of TFA and MSA, and then stirred for 20 min. The PBONF solution was obtained. CNTs (0.1 wt %) and as-synthetic PBONF solution were mixed for 1 hour to prepare CNT/PBONF hybrid dispersion. The CNT/PBONF dispersion was further exposed to ultrasonic treatment for 1 hour. At the same time, a PVA solution (3 wt%) was prepared by stirring for 2 h at 90 °C. The PBONF-reinforced CNT layer was prepared by vacuum filtration of 20 mL CNT/PBONF dispersion through a polytetrafluoroethylene filter membrane with the pore diameter of 0.45 μm and subsequent washing by ethanol for 3 times. 10 mL PVA solution was then filtrated, and another 5 mL PVA solution was casted on the surface of the film. The PBONF-reinforced CNT/PVA bilayer actuator was obtained after air drying for 36 h at room temperature. The actuator was dipped into the water for 10 min for several times to remove the residual acids, and then dried under natural air. The saturated aqueous solutions of CaCl_2_, CH_3_COOK, K_2_CO_3_, NaBr, NaCl, KCl, and K_2_SO_4_ in a closed glass container at 20 °C were used to obtain environments with the specific humidity, which yielded RH of approximately 16.8, 23, 44, 58, 75, 86, and 98%, respectively.

### Characterization of PBONF-reinforced CNT/PVA bilayer actuators

The morphologies of the PBONF-reinforced CNT/PVA bilayer actuators were characterized by SEM (Helios Nanolab 600i, USA). A transmission electron microscopy (TECNAIF20) was used to characterize the morphologies of PBONFs. A semiconductor tester (Keithley 4200-SCS) was used to test the electric conductance of PBONF-reinforced CNT layers, PVA layers, and CNT/PVA bilayers. An Olympus BX53 fluorescence microscope equipped with a 40x objective and the relative software was employed to record the thickness change of the PVA layers. To facilitate the measurement of the thickness changing, we chose a bilayer actuator in which the PVA layer has the thickness of ca. 42 μm at the RH of 23%. The mechanical properties of the freestanding PBONF-reinforced CNT/PVA bilayer actuator was measured in the tensile mode by using a universal mechanical testing machine (Instron 5969, USA). The tested rectangular strips (18 mm × 5 mm) were cut out from a freestanding bilayer actuator with a scalpel. The distance between the clamps was 10 mm and the load speed was 5 mm min^−1^. The RH of the test environment is 32%. A home-built atomic force microscope (AFM)^61^ was used for detecting the compressive modulus of PVA layers at different RHs.

### Theoretical simulation

The simulation of the bending of the PBONF-reinforced CNT/PVA bilayer actuators was done by using the finite element simulation, and a bilayer structure is adopted to model the PBONF-reinforced CNT/PVA bilayers with the mechanical and swelling properties according to the experimental data. The software COMSOL Multiphysics 4.3a (COMSOL, Stockholm, Sweden) which is based on a finite element algorithm was chosen for simulating, whereby the thermoelasticity plug-in was used. The humidity-based swelling was approximated by utilizing the corresponding thermal expansion coefficients in the customized materials and by adjusting the virtual temperature.

## Electronic supplementary material

Polybenzoxazole Nanofiber-Reinforced Moisture-Responsive Soft Actuators
