# Supplementary material for: Polybenzoxazole Nanofiber-Reinforced Moisture-Responsive Soft Actuators
Source: Sci Rep. 2017 Apr 10;7:769. doi: 10.1038/s41598-017-00870-w (PMC5429722; doi:10.1038/s41598-017-00870-w)
Supplement: Supplementary file 1 — Polybenzoxazole Nanofiber-Reinforced Moisture-Responsive Soft Actuators [file 41598_2017_870_MOESM1_ESM.doc]

Supplementary Information

Polybenzoxazole Nanofiber-Reinforced Moisture-Responsive Soft Actuators

**Meiling Chen^1^,** **Johannes Frueh^1^,** **Daolin Wang^1^,** **Xiankun Lin^1,*^,** **Hui Xie^1,2,*^,** **and Qiang He^1,*^**

^1^ Key Laboratory of Microsystems and Microstructures Manufacturing, Ministry of Education, Micro/Nanotechnology Research Centre, Harbin Institute of Technology, Harbin, 150080, China

^2^ State Key Laboratory of Robotics and Systems, School of Mechatronics Engineering, Harbin Institute of Technology, Harbin, 150080, China

*[xiankunlin@hit.edu.cn](mailto:xiankunlin@hit.edu.cn); xiehui@hit.edu.cn; [qianghe@hit.edu.cn](mailto:qianghe@hit.edu.cn)


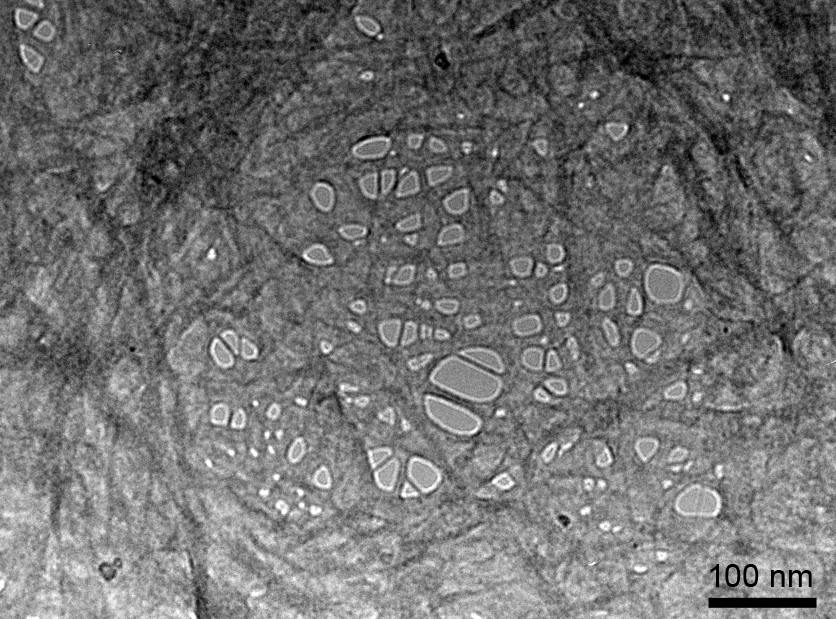


**Figure S1.** Transmission electron microscopic image of PBONFs.


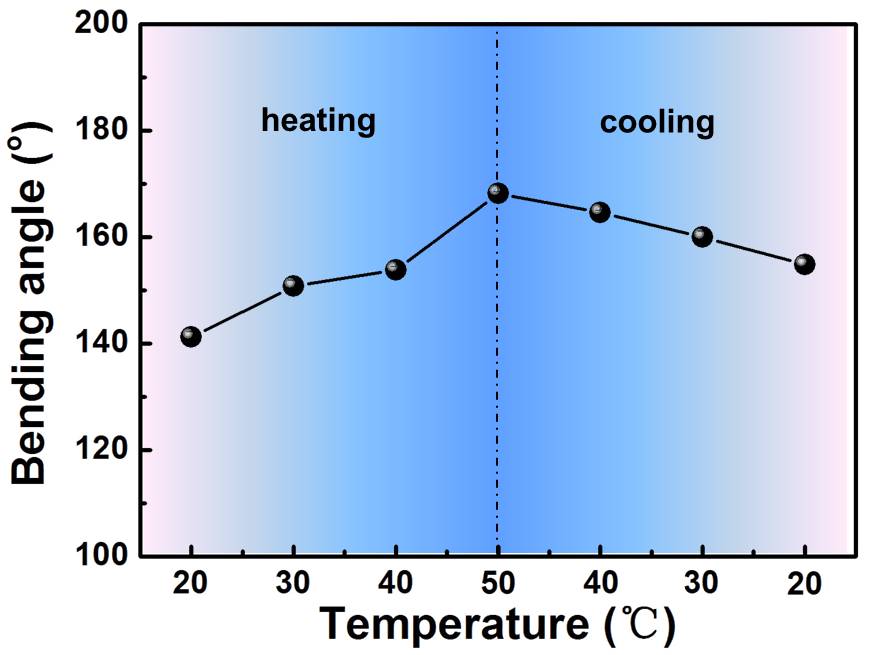


**Figure S2.** The influence of temperature on the bending angle of the PBONF-reinforced CNT/PVA bilayer actuators at the RH of 30%.


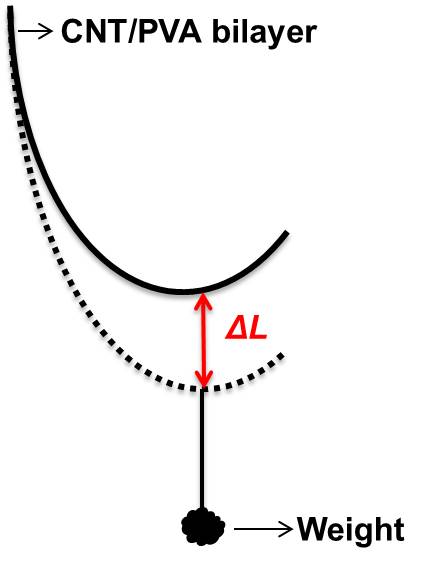


**Figure S3.** Scheme showing the moving distance of the actuator (*ΔL*) caused by a weight.


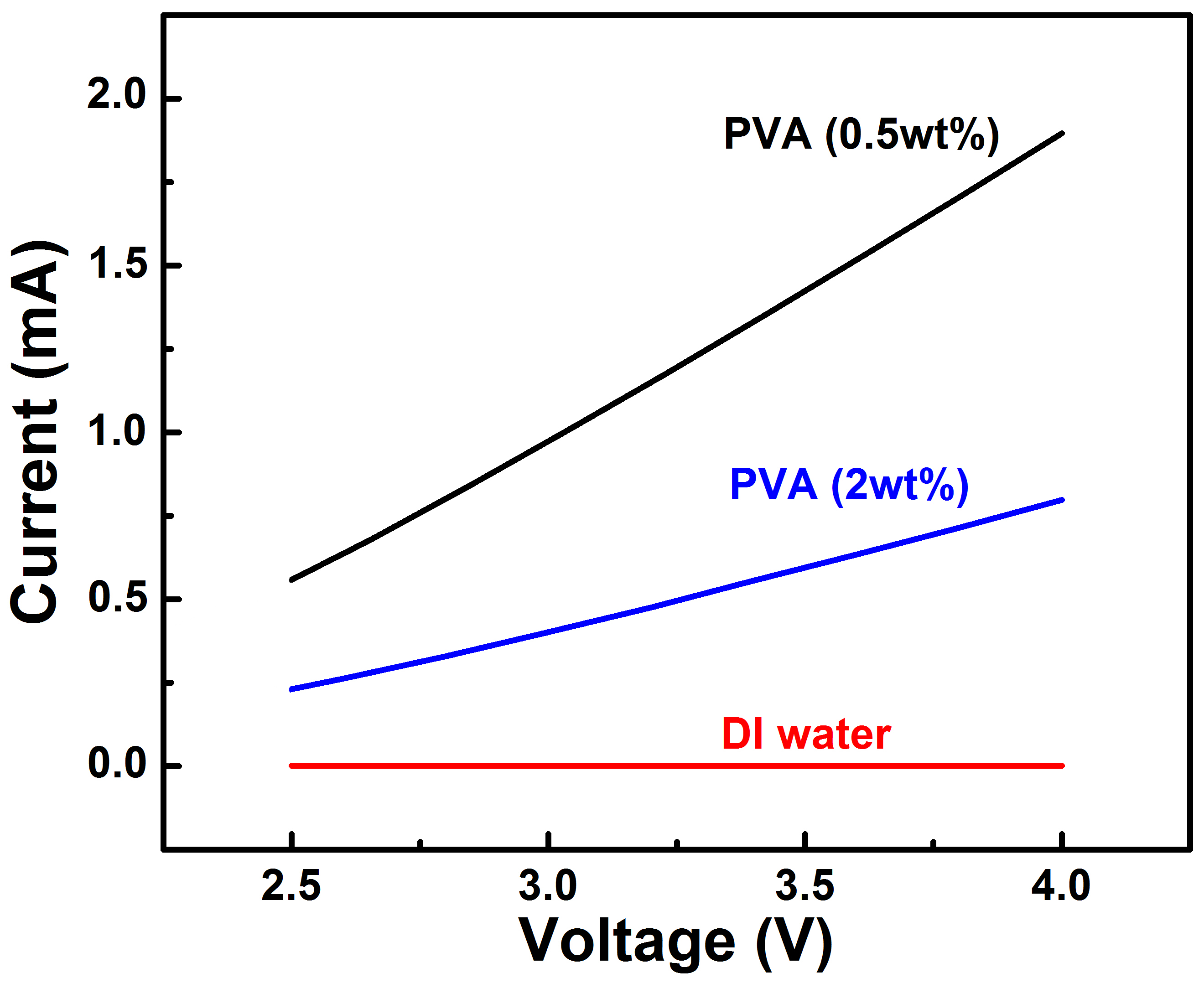


**Figure S4.** Current–voltage (I–V) characteristics of [deionized](../../../../Documents%20and%20Settings/Administrator/Local%20Settings/Application%20Data/Yodao/DeskDict/frame/20150405091509/javascript:void(0)%3B) [water](../../../../Documents%20and%20Settings/Administrator/Local%20Settings/Application%20Data/Yodao/DeskDict/frame/20150405091509/javascript:void(0)%3B) and the PVA solutions with the concerntrations of 0.5 wt% and 2 wt%.


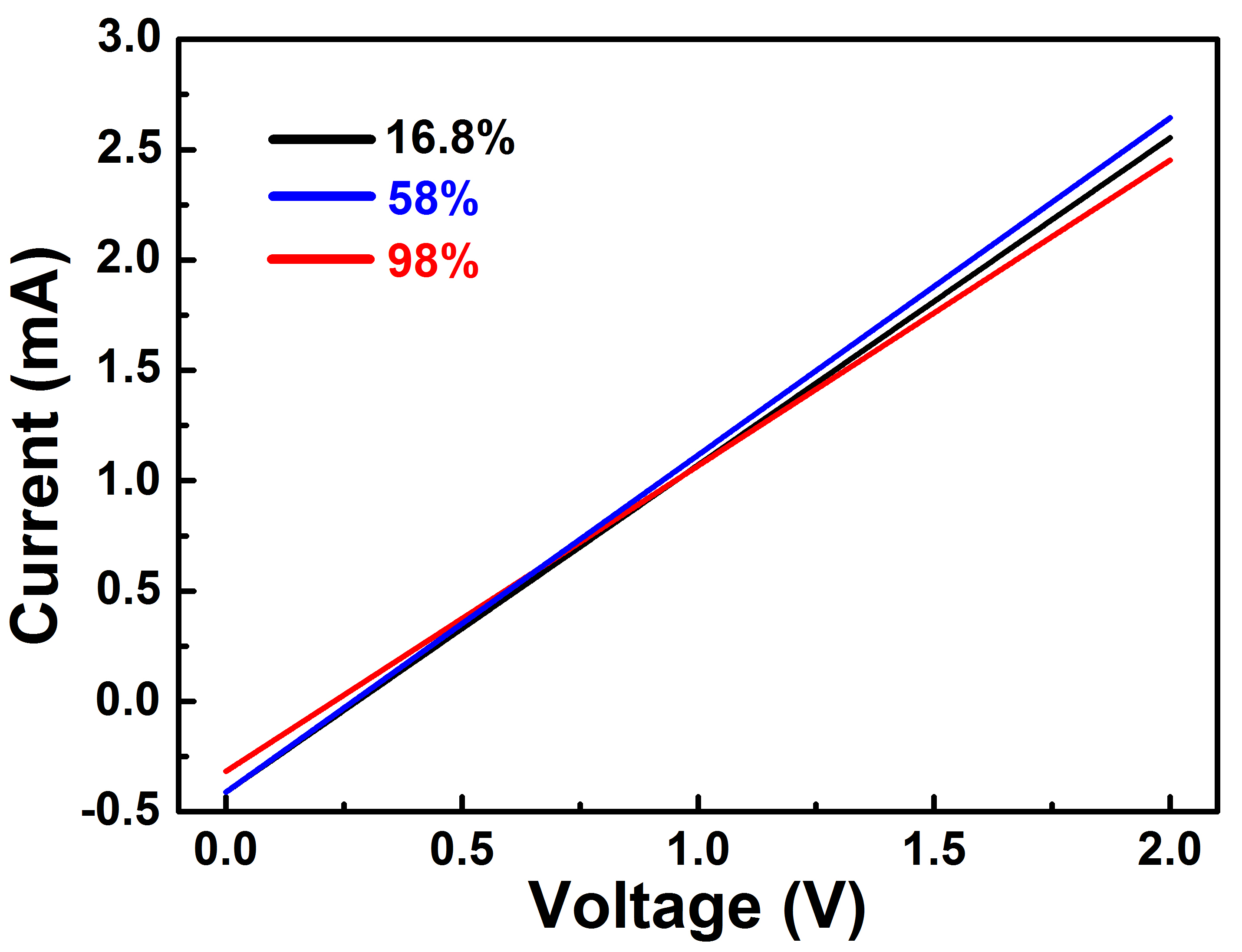


**Figure S5.** Current–voltage (I–V) characteristics of the PBONF-reinforced CNT/PVA bilayer at the RHs: 16.8% (black line), 58% (blue line), and 98% (red line). The bilayer was forced to be unbending by fixing the ends when the RH changed.


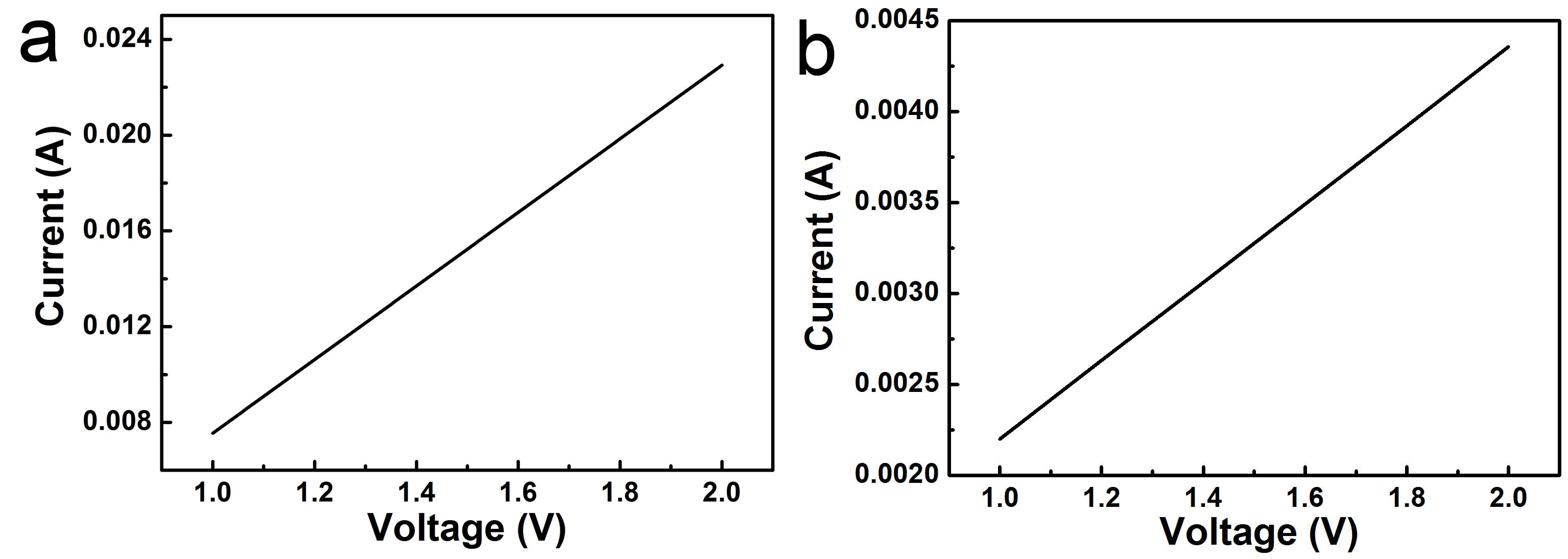


**Figure S6.** Current–voltage (I–V) characteristics of (a) a pristine CNT layer and (b) a PBONF-reinforced CNT layer at the RH of 30%.

The pristine CNT layer (10 mm × 10 mm) with the thickness of ca. 85 μm was prepared by filtrating through a PTFE filter membrane. The conductivity of the pristine CNT layer was calculated to be 1.81 S/cm, while the conductivity of the PBONF-reinforced CNT layer with the dimension of 7.42 mm × 3.60 mm × 26.5 μm (length × width × thickness) is 1.67 S/cm.
